# Supplementary material for: The impact of green technology innovation on carbon emission reduction capacity in China: Based on spatial econometrics and threshold effect analysis
Source: PLoS One. 2024 Dec 11;19(12):e0310208. doi: 10.1371/journal.pone.0310208 (PMC11634009; doi:10.1371/journal.pone.0310208)
Supplement: Attachment — Submitted filename: Response to Reviewers.docx [file pone.0310208.s001.docx]

**Reviewers' comments:**

**Reviewer #1:**

It is my pleasure to review the current manuscript having title “The Impact of Green Technology Innovation on Carbon Emission Reduction Capacity in China: Based on Spatial Econometrics and Threshold Effect Analysis” for the esteemed journal. The manuscript has some merits but there are certain issues which need to be addressed to improve the quality of the manuscript. The authors must consider the following suggestions:

1. The abstract is well-structured. However, it should further underscore the scientific value added of your paper in your abstract.

Reply: The analysis of the internal mechanism of green technology innovation affecting carbon emission reduction will help provide the development direction for green technology innovation, help China's carbon neutrality and carbon peak goals as soon as possible, and contribute Chinese wisdom to the sustainable development of the world.The analysis of the internal mechanism of green technology innovation affecting carbon emission reduction will help provide the development direction for green technology innovation, help China's carbon neutrality and carbon peak goals as soon as possible, and contribute Chinese wisdom to the sustainable development of the world.

2. The novelty of this paper should be further justified by highlighting main contributions of the paper.

Reply: First, from the research perspective, this paper earlier research green technology innovation affect carbon reduction ability, green provide new application direction for green science and technology innovation, a few research focus on green technology innovation of carbon reduction effect, but focus on carbon emissions as a research object, has certain limitations, this paper on carbon emission reduction capacity, better reflect the green technology innovation on the ability of carbon emission reduction, more research value; Second, from the perspective of research methods, the spatial effect model and panel threshold model are used to conduct empirical analysis of 30 provinces in China to investigate the direct effect, spillover effect and threshold characteristics of green technology innovation on carbon emission reduction capacity; Third, from the perspective of research significance, it is of great significance to clarify the mechanism effect of the regional carbon emission reduction capacity, which is of great significance for promoting the comprehensive transformation of economic and social development and the harmonious coexistence between man and nature.

3. More recent and relevant papers should be cited to enrich the literature.

Reply: About 10 articles on the relationship between green technology innovation and carbon emission reduction in the past three years have been added.

4. In the methodology section, the model of the study needs to be justified by comparing it with other models.

Reply: Advantages of the spatial Durbin model: Advantages of the spatial Durbin model: Compared with other models of spatial metrology models, SDM models are able to capture both spatial lag and spatial error terms, it not only has the characteristics of the general spatial measurement model, but also can decompose the direct effects and indirect effects from the total effects.

Rationsibility of the panel threshold model: Threshold studies mainly include group test, cross-term test and panel threshold model. Group test relies on artificial setting of segmentation points, with strong subjectivity. Cross-term test has uncertainty, and the significance of the estimated results is difficult to solve. The panel threshold model makes up for the shortcomings of the above methods by using the estimated threshold parameter as a segment function, which can automatically identify the mutation points of the data and conduct the significance test.

5. In the results section, the obtained results should be compared with existing studies in the field.

Reply: In section 5.4.3 Analysis results of SDM and 5.5.3 Results analysis of threshold regression model, the comparison with existing studies is added, and the literature numbers are 52,53,54,55, which confirms the conclusion of this paper, and the robustness test also proves that the conclusion is more reliable.

6. Grammar check is required to avoid any possible English errors.

Reply: We have touched my native English speakers.

**Reviewer #2:**

1. Thank you for submitting your manuscript, "The Impact of Green Technology Innovation on Carbon Emission Reduction Capacity in China: Based on Spatial Econometrics and Threshold Effect Analysis," to our journal. This topic is crucial and timely, particularly in the context of global efforts to reduce carbon emissions. Your methodological choice of spatial econometrics and threshold effect analysis is robust and suitable. However, several areas require enhancement to clarify, deepen, and empirically support your manuscript more effectively.

Reply: We has been modified according to your opinion.

2. The abstract should more distinctly articulate your unique contributions, setting your work apart from existing studies. In the introduction, explicitly state your research questions and objectives rather than implying them to guide your readers more clearly. Your literature review should extend beyond Chinese studies to include global contexts, enhancing the applicability and depth of your analysis. More critical engagement with the existing literature, highlighting limitations and controversies, will solidify your study's foundation.

Reply: Unique contribution: This study innovatively adds the carbon market vitality in measuring carbon reduction capacity, and the study extends the previous carbon reduction capacity to expand the influence of green technology innovation and is more practical, and explores the relationship between green technology innovation and carbon reduction capacity.

3. Your theoretical framework and hypotheses are promising but need clearer explanations of mechanisms like spatial spillover effects and non-linear characteristics within green technology contexts. Definitions for key terms and variables, such as "green technology innovation," must be precise to maintain scientific rigor.

Reply: The literature review section was refined, some scholars have ignored the fact that the meaning of carbon emission reduction has changed. Compared with carbon emission reduction, carbon emission reduction capacity is sustainable and can provide reference for the world.

Lack of regional integration and even global integration development pattern thinking.

4. Your methodology should include detailed descriptions of your data collection processes, sources, and integrity measures. Clarify how key variables are measured and operationalized and provide detailed justifications for your chosen statistical methods and models, including how model parameters were estimated and model robustness assessed.

Reply: **The study methodology section** incorporates the data collection process, this paper takes Chinese provinces as the research object to study the relationship between green science and technology innovation and carbon emission reduction capacity. Select the macro data of each province. The government agencies collect a large amount of data in the process of performing their duties, and this paper obtains through the government open data platform, public database and other ways. Details are as follows:

Compared with other models, the spatial Dubin model and the panel threshold model have their own unique advantages.

**Advantages of the spatial Durbin model**: Advantages of the spatial Durbin model: with other models of spatial metrology models, SDM models are able to capture both spatial lag and spatial error terms, it not only has the characteristics of the general spatial measurement model, but also can decompose the direct effects and indirect effects from the total effects.

**Rationsibility of the panel threshold model**: Threshold studies mainly include group test, cross-term test and panel threshold model. Group test relies on artificial setting of segmentation points, with strong subjectivity. Cross-term test has uncertainty, and the significance of the estimated results is difficult to solve. The panel threshold model makes up for the shortcomings of the above methods by using the estimated threshold parameter as a segment function, which can automatically identify the mutation points of the data and conduct the significance test.

**The robustness test** was performed to exclude the possibility that the study results were not completely correct. Replacement variables are one of the most common robustness test methods. Different ways of measuring variables may lead to deviation in the results. Spatial measurement model is sensitive to the setting of the relationship of spatial weight matrix, and different matrices may also lead to deviation in the results.

5. Address whether appropriate controls were implemented during your analyses and describe any replication measures or sensitivity analyses conducted to confirm the robustness and reliability of your results.

Reply: In order to confirm the robustness of the results, the core explanatory variables and spatial weight matrix are replaced to verify the reliability of the direct and indirect effects of green technology innovation on carbon emission reduction capacity(Section 5.4.4); and the added control variable method is adopted to verify the reliability of the increasing marginal effect of green technology innovation on carbon emission reduction capacity(Section 5.4.4)
